# Differential expression of galanin in the cholinergic basal forebrain of patients with Lewy body disorders

**DOI:** 10.1186/s40478-015-0249-4

**Published:** 2015-12-01

**Authors:** Athanasios Alexandris, Alan King Lun Liu, Raymond Chuen-Chung Chang, Ronald K. B. Pearce, Steve M. Gentleman

**Affiliations:** Division of Brain Sciences, Department of Medicine, Imperial College London, Burlington Danes Building, Hammersmith Hospital Campus, London, W12 0NN UK; School of Medicine, University of Leicester, Leicester, UK; Laboratory of Neurodegenerative Diseases, School of Biomedical Sciences, LKS Faculty of Medicine, The University of Hong Kong, Pokfulam, Hong Kong SAR; Research Centre of Heart, Brain, Hormone, and Healthy Aging, LKS Faculty of Medicine, The University of Hong Kong, Pokfulam, Hong Kong SAR; State Key Laboratory of Brain and Cognitive Sciences, The University of Hong Kong, Pokfulam, Hong Kong SAR

## Abstract

**Introduction:**

Depletion of cholinergic neurons within the nucleus basalis of Meynert (nbM) is thought to contribute to the development of cognitive impairments in both Alzheimer’s disease (AD) and Lewy body disorders (LBD). It has been reported that, in late stage AD, a network of fibres that contain the neuropeptide galanin displays significant hypertrophy and ‘hyperinnervates’ the surviving cholinergic neurons. Galanin is considered as a highly inducible neuroprotective factor and in AD this is assumed to be part of a protective tissue response. The aim of this study was to determine if a similar galanin upregulation is present in the nbM in post-mortem tissue from patients with LBD. Gallatin immunohistochemistry was carried out on anterior nbM sections from 76 LBD cases (27 PD, 15 PD with mild cognitive impairment (MCI), 34 PD with dementia (PDD) and 4 aged-matched controls. Galaninergic innervation of cholinergic neurons was assessed on a semi-quantitative scale.

**Results:**

The LBD group had significantly higher galaninergic innervation scores (*p* = 0.016) compared to controls. However, this difference was due to increased innervation density only in a subgroup of LBD cases and this correlated positively with choline acetyltransferase–immunopositive neuron density.

**Conclusion:**

Galanin upregulation within the basal forebrain cholinergic system in LBD, similar to that seen in AD, may represent an intrinsic adaptive response to neurodegeneration that is consistent with its proposed roles in neurogenesis and neuroprotection.

**Electronic supplementary material:**

The online version of this article (doi:10.1186/s40478-015-0249-4) contains supplementary material, which is available to authorized users.

## Introduction

Cognitive dysfunction has been increasingly recognised as an integral feature of Lewy body disorders (LBD). The severity of cognitive dysfunction and its temporal presentation in relation to Parkinsonian motor symptoms allows the clinical separation of LBD into Parkinson’s disease (PD), Parkinson’s disease with dementia (PDD) and dementia with Lewy bodies (DLB). Although early cognitive deficits in PD may be caused by failure in multiple neurotransmitter systems, cholinergic dysfunction seems to play a significant role in the progression to dementia [1]. The severe depletion of the basal forebrain cholinergic neurons in the nucleus basalis of Meynert (nbM) has long been regarded as a key neuropathological substrate for cognitive impairment in Alzheimer’s disease (AD) and LBD [2].

The early vulnerability of the cholinergic system and other neurotransmitter systems arising from several subcortical nuclei of reticular neurons -the isodendritic core complex- in LBDs and other dementias [3–6] remains largely unexplained. However, it was recognised very early that degeneration of these systems is associated with significant plasticity of the surviving neurons [3]. In 1988, Chan-Palay provided preliminary evidence that in several AD cases there was significant hypertrophy of a network of fibres that innervate the basal cholinergic neurons (termed hyperinnervation) and contain the neuropeptide galanin. This was particularly evident in the anterior nbM [7, 8].

Galanin is a pleiotropic neuropeptide that is widely distributed within the human nervous system [9, 10] and exists as either 19 or 30-amino acid long isoforms (in contrast to 29 amino acid long as first extracted from porcine intestines) [11–13]. Galanin is known to participate in the regulation of several neuroendocrine [14] and neurotransmitter systems [15, 16] via three galanin G-protein coupled receptors (GAL1-3) [17]. Current literature suggests that galanin is an important modulator of the basal cholinergic system [18] and so changes in its expression may be relevant to the development of dementia. The underlying aetiology and consequences of galaninergic hyperinnervation of the nbM neurons are not well understood but it is currently thought that galanin upregulation in degenerative tissue may represent a neuroprotective mechanism [19] that could be potentially exploited pharmacologically.

Apart from the descriptive report on galanin hyperinnervation of three PDD cases by Chan-Palay [7], there has not been any investigation of the expression of galanin using contemporary definitions of PD and PDD, without concurrent AD.

Here, we hypothesise that the extent of galaninergic innervation within the nbM will increase through the progression from controls to PD to PDD. The specific aims of this study were to characterise galaninergic innervation and expression pattern within the basal forebrain region, and to investigate whether the patterns of innervation differ between PD and PDD.

## Materials and methods

### Case selection

A total of 177 cases were identified and reviewed from the Parkinson’s UK Brain Bank at Imperial College London. Seventy-six LBD cases along with 4 age-matched controls were selected based on tissue availability. Retrospective case-note analysis using clinical summaries compiled from hospital and GP records by movement disorder specialists and neurologists was undertaken to classify LBD cases into PD without cognitive impairment, PD with mild cognitive impairment (PD-MCI) and PDD. PD was defined neuropathologically according to consensus neuropathological criteria [20] and clinically by presence of 2 out of 4 cardinal motor symptoms (resting tremor, rigidity, hypokinesia and postural instability) [21]. Based on clinical summaries and neuropsychological assessments (if available), patients with PD who develop cognitive deficits severe enough to interfere with independent activities of daily living, satisfying DSM-IV and ICD-10 clinical criteria for dementia and Movement Disorder Society Task Force diagnostic criteria for PDD [22], were classified as PDD on the condition that this developed more than one year after Parkinsonism symptoms. PD-MCI was defined as a decline in one or more domains of cognitive function without significant impairment in the activity of daily living of the patient [22]. For our exclusion criteria, cases with poor or incomplete clinical notes and subjects with last clinician visit more than 2 years prior death were not included in the present study. Any cases with co-existing AD pathology (Braak & Braak tau stage 4 or above), cerebral vascular pathologies and other co-existing neuropathological diagnosis were excluded. Poorly fixed and long post-mortem delay of tissue (>72 h) which may impact on the quality of subsequent immunostaining were also excluded. In total, 27 cases of PD without cognitive impairment, 15 cases of PD-MCI and 34 cases of PDD were selected for this study. Retrospective case-note analysis is a well-accepted method of case ascertainment and has often been used in clinic-pathological studies on Lewy body disorders [23–25].

### Definition of the anterior nbM subregion

**Fig. 1:**
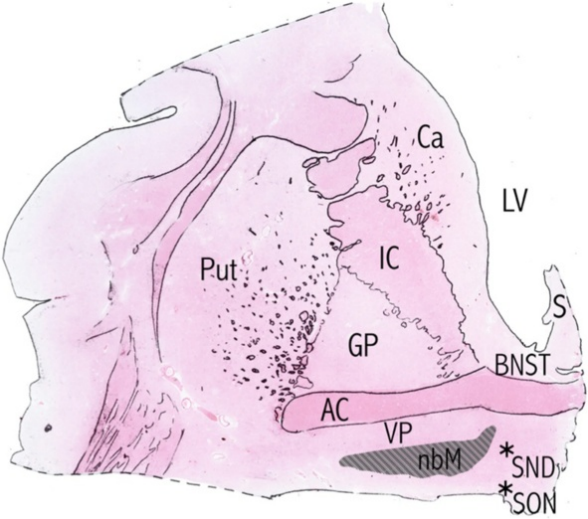
Drawing of a H&E-stained section through the anterior nucleus basalis of Meynert. *AC* anterior commissure, *BNST* bed nucleus of stria terminalis, *Ca* caudate nucleus, *GPe*/*i* globus pallidus externus/internus, *IC* internal capsule, *LV* lateral ventricle, *nbM* nucleus basalis of Meynert (*grey* area represents expected distribution), *Put* putamen, *SND* sexually dimorphic nucleus of the preoptic area, *S* septum, *SON* supraoptic nucleus

Following standard protocol, formalin-fixed paraffin-embedded (FFPE) basal forebrain specimens including the nbM were cut in the coronal plane in 7 μm thick sections. The identification of the anterior nbM subregion was based on our previously defined boundaries [2] situated at the level of the decussation of the anterior commissure and dorsal-lateral to the supraoptic nucleus (Fig. 1). Magnocellular neurons medial to the supraoptic neurons were considered part of the Ch2 diagonal band nucleus.

The selection of the anterior nbM region for this study is justified given that a) the degeneration of nbM neurons in this area has been well characterised in both AD and LBD and b) galanin fibres are more pronounced in the anterior basal forebrain and anterior nbM compared to more posterior regions in both health and disease [9, 26].

### Galanin immunohistochemistry

FFPE Sections were first dewaxed and rehydrated by sequential immersion for 2 × 5 min in xylene and decreasing concentrations of industrial methylated spirit (IMS; 100, 100, 90, 70 %) and distilled water (dH_2_O). Endogenous peroxidase activity was blocked in 1 % H_2_O_2_ in phosphate-buffered saline (PBS, pH 7.4) for 30 min. Antigen retrieval was achieved using a steamer (20 min) in 0.01 M trisodium citrate buffer (pH 6.0). Sections were then immersed in dΗ2Ο and in PBS (3 × 5 min) before incubation with a monoclonal galanin antibody raised against the Ala20-Ser123 peptide portion of human galanin (1:7000, R&D Systems, MAB585) overnight at 4 °C (See Additional file 1 for peptide sequence).

On the second day, sections were first immersed in PBS (2 × 5 min). Sections were visualised with the SuperSensitive Link-Label Immunohistochemistry Detection System (BioGenex, UK) with 3′3-diaminobenzidine (DAB) according to manufacturer’s manual. All sections were counterstained with Mayer’s haematoxylin and dehydrated in increasing concentration of IMS (70, 90, 100, 100 %) and xylene (2×) before coverslipping with DPX (Distrene, Plasticiser, Xylene).

Positive controls for the validation of galanin antibodies were sections including hypothalamic nuclei [9, 10, 27]. The omission of primary antibodies was used as negative control. The specificity of the galanin antibody was further investigated with pepsin pretreatment which digests peptides but not lipofuscin [28]. All negative controls showed no specific galanin immunoreactivity (GAL-ir). Staining patterns were also compared with immunostaining with a commercial polyclonal antibody raised against the His51-Lys63 sequence of the human galanin (See Additional file 1).

However, the potential for cross-reactivity with other similar antigens cannot be excluded and hence GAL-ir is regarded as GAL-*like* immunoreactivity.

### Double immunofluorescence of galanin and GFAP

FFPE Sections were dewaxed, rehydrated and pre-treated as described above. Sections were then incubated with mouse monoclonal anti-galanin antibody (1:1000, R&D Systems, MAB585) and rabbit polyclonal anti-glial fibrillary acidic protein (GFAP, 1:500, Dako, Z0334) diluted in PBS with 2 % goat serum and 0.3 % Triton-X 100 overnight at 4 °C. On the second day, sections were first immersed in PBS (2 × 5 min), then incubated with goat-anti-mouse secondary antibody conjugated with Alexa Fluor® 568 fluorophore (1:200, ThermoFisher Scientific, A-11004) and goat-anti-rabbit secondary antibody conjugated with Alexa Fluor® 488 fluorophore (1:200, ThermoFisher Scientific, A-11008) for 1 h at room temperature. Sections were then rinsed briefly in PBS (3 × 5 min) and incubated in 1 % sudan black B dissolved in 70 % ethanol to block endogenous autofluorescence by lipofuscin, before coverslipping and mounting with VECTASHEILD antifade mounting medium with DAPI (Vector Laboratories, UK).

### Semi-quantitative scoring of galaninergic innervation

**Fig. 2:**
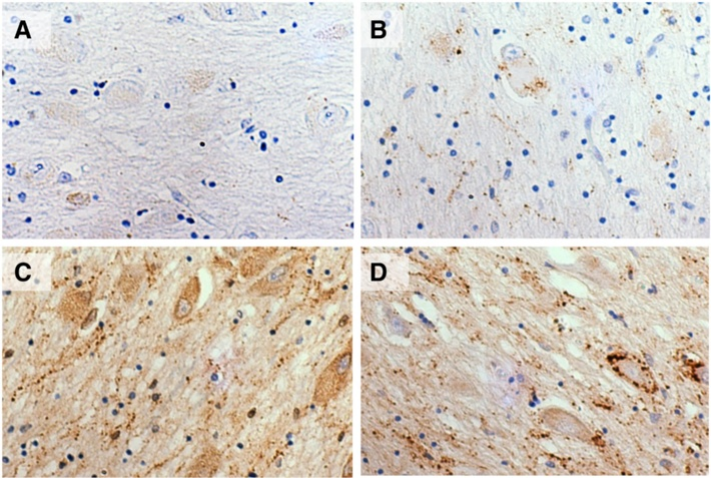
Galanin innervation semi-quantitative scale. **a** Grade 0, no visible innervation. **b** Grade 1, minimal innervation. Fibres in the neuropil are sparse. **c** Grade 2, moderate innervation. Many thick fibres in the neuropil and several contacts with somata/dendrites. **d** Grade 3, Hyperinnervation. Abundant hypertrophic fibres in the neuropil with frequent contacts with neurons

As reported in the literature, the absolute quantification of GAL-ir terminals and innervation is technically difficult [7, 26, 29, 30]. Hence a semi-quantitative scale with four grades was devised (Fig. 2). All sections were stained with a monoclonal antibody against galanin. A photomicrograph of the area of maximal innervation within the limits of the anterior nbM from each stained section were captured on an Olympus AHBT3 VANOX microscope with digital camera at ×200 magnification. The images were individually graded according to the semi-quantitative scale by two independent assessors (AA, AKLL) blinded to diagnosis. For inter-rater reliability, Cohen’s *κ* = 0.86 and weighted *κ* = 0.92 (SE of κ = 0.13). For intra-rater reliability, Cronbach’s *α* = 0.98. These reliability coefficient scores indicate an excellent inter- and intra-rater reproducibility of the semi-quantitative grading.

### Confocal microscopy

Imaging of double immunofluorescence-stained tissues was performed using a Zeiss LSM-780 inverted confocal laser scanning microscopes (Carl Zeiss, Germany) at the Facility for Imaging by Light Microscopy (FILM) facility in Hammersmith Hospital. A ×10 objective (EC Plan-Neofluar, numerical aperture, 0.3; working distance, 5.2 mm) and ×20 objective (Plan Apochromat DIC, numerical aperture, 0.8; working distance, 0.55 mm) with laser excitation at 405, 488, 543 and 594 nm were used. Image capturing and processing were performed using the Zen Black (Carl Zeiss, Germany) software.

### Statistical analysis

Demographic characteristics were tested for normality with the Shapiro-Wilk test and visual inspection of Q-Q plots, and then compared with one-way ANOVA (*F*). The Mann-Witney test (U) was used to test the one-tailed hypothesis that disease cases will have increased innervation compared to controls. The Kruskal-Wallis test (*H*) with post-hoc pairwise comparison was used for analysis of innervation scores among different diagnostic groups. Spearman rank correlation (rho) was used for non-parametric analysis of associations. Statistical significance was set at *p* < 0.05. Non-adjusted p-values are shown. Statistical analyses were performed on IBM Statistical Package for Social Sciences software (SPSS v22) and GraphPad Prism 6 software.

## Results

### Cohort characteristics

For the 3 cohorts included in this study (Table 1) the age at disease onset, age at death and sample region, did not differ significantly between groups, although most of them were male due to tissue availability limitations. Argyrophilic grain pathology is very common and was found in one PDD and one PD-MCI case but it is not thought to represent a separate pathological/nosological entity [31]. Control cases had no significant α-synuclein or tau pathology.

**Table 1:** Summary of clinical characteristics. See text for discussion

	Mean age at onset (SD)	Mean age at death (SD)	Mean duration of disease (SD)	Median Braak α-synuclein stage	Median Braak tau stage	% of Males
CONTROLS (*n* = 4)	–	82.75 (5.11)	–	0	2	25.0
PD (*n* = 27)	66.59 (8.58)	77.48 (7.15)	10.93 (6.01)	6	2	55.6
PD-MCI (*n* = 15)	60.93 (9.95)	75.20 (8.45)	14.40 (6.31)	6	1	53.3
PDD (*n* = 34)	63.88 (10.31)	77.12 (8.13)	13.35 (5.58)	6	2	70.6

### General patterns of galanin-like immunoreactivity 

**Fig. 3:**
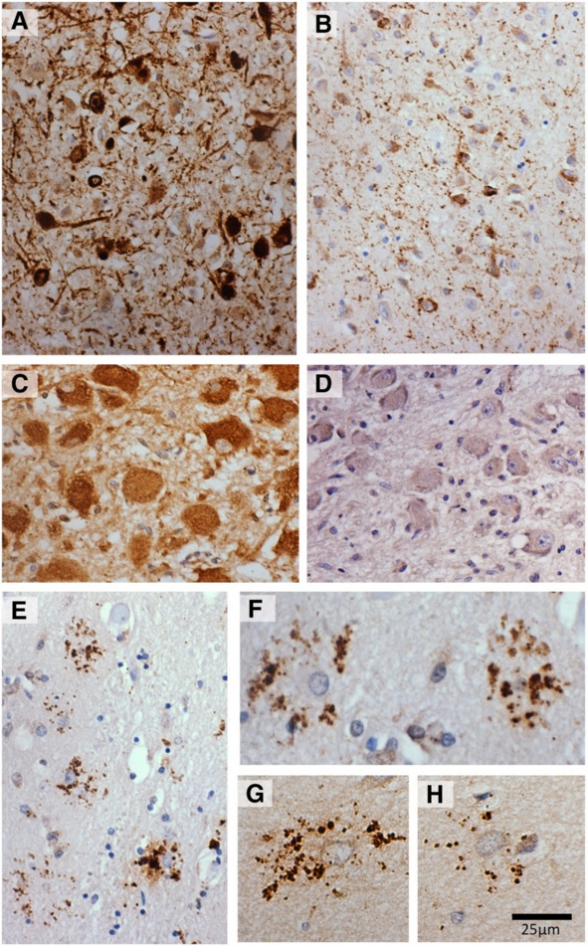
Different patterns of galanin immunoreactivity in the basal forebrain. **a** Intensely immunoreactive neurons of the hypothalamic nuclei. **b** Galanin immunoreactive neurons and fibres in the sexually dimorphic nucleus of the preoptic area, also known as intermediate hypothalamic nucleus (20×). **c**-**d** Spectrum of perikaryal immunoreactivity within the neurons of the supraoptic nucleus. **e**-**h** putative glia with GAL-ir of radial morphology found within the ventral pallidum (E captured at 20×; F-H captured at 40×; *scale*-*bar* for F-H)

In accordance with observations made previously [8–10, 26, 27] intensely stained bipolar and multipolar neurons were observed in the medial and lateral hypothalamus (Fig. 3a) in both control and disease cases. A few intensely GAL-ir parvicellular neurons and dense fibres were also observed in the neighbouring sexually dimorphic nucleus of the preoptic area (intermediate nucleus; Fig. 3b), already known to be galaninergic [32].

Galanin-immunoreactive fibres were intensely stained and widely distributed in all sections. A high density of galaninergic fibres dorsomedial to nbM was always present and in line with a proposed galaninergic pathway that courses through the substantia innominata (sub-commissural region) en route to the hypothalamus, bed nucleus of the stria terminalis and the nucleus of the vertical limb of diagonal band (Ch2) [8, 9].

**Fig. 4:**
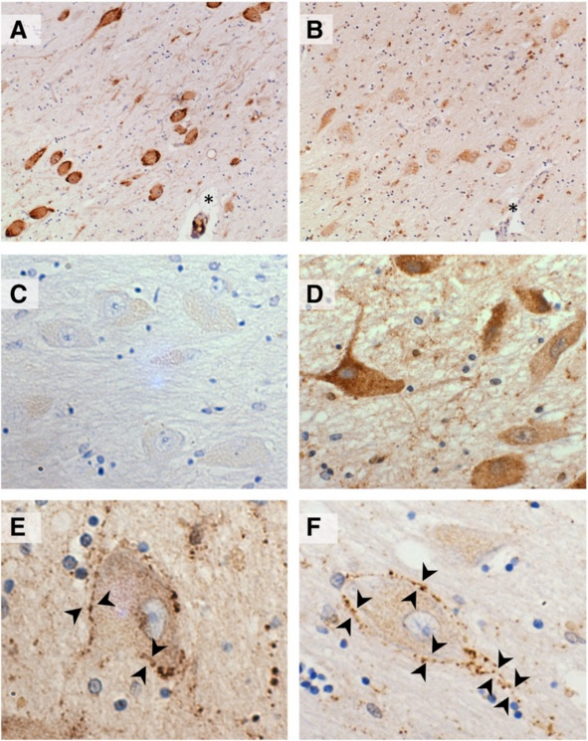
Galanin like immunoreactivity (GAL-ir) of putative cholinergic neurons. **a**-**b** Sections of ChAT (**a**) and galanin (**b**) immunostaining of the nucleus basalis magnocellular neurons from the same case; * denotes same anatomical landmark. **c** Example of minimal somal immunoreactity. **d** Example of intense perikaryal GAL-ir. **e**-**f** Galaninergic innervation of putative cholinergic neurons (*arrow heads*)

**Fig. 5:**
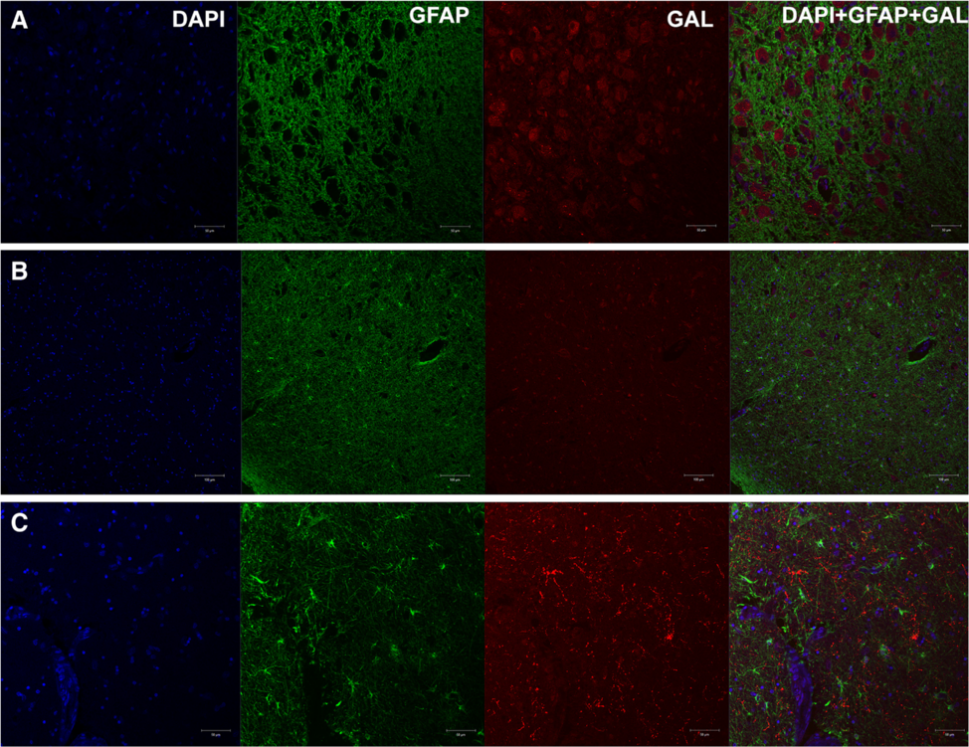
GFAP and galanin immunofluorescence with confocal microscopy. **a** Supraoptic nucleus showing galanin immunofluorescence of perikarya (×10) **b** Nucleus basalis of Meynert showing putative cholinergic neurons with perikaryal galanin immunofluorescence. **c** Ventral pallidum showing lack of association between glial galanin-like immunoreactivity and the astrocytic marker GFAP (×20)

Intense somal GAL-ir was observed in the hypothalamic nuclei (Fig. 3a-b) and the supraoptic nucleus (SON) exhibited variable levels of perikaryal staining and very few fibres (Fig. 3c-d). Some somal GAL-ir was observed in the nbM and sections from the same case immunostained with galanin and ChAT antibodies reveal immunoreactivity of the same magnocellular cell population (Fig. 4a-b). It was noticed that there was no concordance between perikaryal staining intensity in the SON and that of the neighbouring nbM neurons. Confocal microscopy demonstrated low level galanin immunofluorescence in both SON and nbM magnocellular neurons (Fig. 5a-b).

Finally, GAL-ir of a radial morphology (25–50 μm in diameter) consistently with a putative glial cell nucleus at the epicentre (10–15 μm), was observed predominately dorso-laterally to the nbM, within the ventral pallidum (Fig. 2e-h). These putative glial cells with GAL-ir were observed in about half of the disease cases and in none of the controls, being more frequent and more prominent in PDD and less so in PD-MCI and PD cases. GFAP immunostaining of adjacent slides and confocal microscopy revealed no co-localization of galanin with the astrocytic marker (Fig. 5c).

### Galaninergic innervation of putative cholinergic neurons

GAL-ir fibres were nearly always visualised within the nbM although their density was very variable. GAL-ir fibres were found to course through the neuropil and to decorate the somata and the dendritic tree of putative cholinergic neurons to different extents (Fig. 2). The very close apposition between the fibres’ varicosities and the magnocellular cell bodies and proximal dendrites suggests the existence of synaptic contacts (Fig. 4d-e). However, as the distal dendritic tree of the magnocellular neurons was not visible we cannot exclude further contacts with the ‘free’ galanin fibres. Direct apposition between fibres and neurons has been demonstrated previously with confocal microscopy [29] while synaptic contacts between galanin-positive fibres and choline acetyltransferase (ChAT)-positive neurons have been characterised in the rat using electron microscopy [33]. In a subgroup of disease-cases there was profound hypertrophy of the galaninergic fibre network in terms of increased fibre density and varicosities and increased perikaryal decoration, similar to the hyper-innervation pattern described previously in AD [7, 8, 29, 30].

**Fig. 6:**
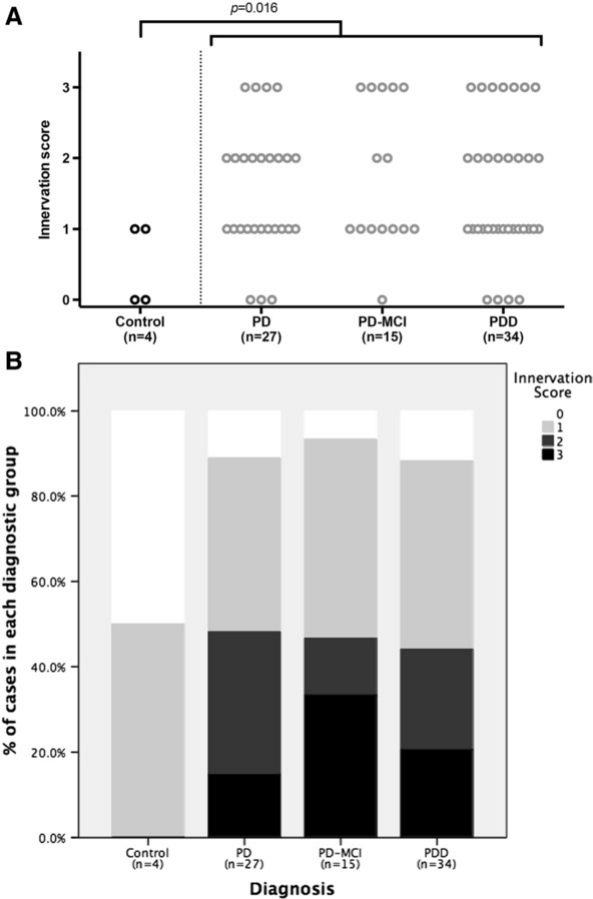
**a** Semiquantitative assessment of galaninergic innervation of nucleus basalis of Meynert in Parkinson’s disease (PD) without or with mild cognitive impairment (PD-MCI), Parkinson’s disease dementia (PDD), and age-matched controls. Horizontal bars indicate the median value with interquartile range. **b** Percentage of cases displaying different innervation scores within each diagnostic category. For explanation of grading system see text

Semi-quantitative assessment of innervation density revealed that the extent of innervation was significantly higher in LBD (PD, PD-MCI and PDD combined) compared to the age-matched controls; *U*(78) = 57.00, Z = −2.219, exact *p* = 0.016 (one-tailed; see Fig. 6a). Further, analysis indicated no significant differences among the different diagnostic groups although it was noticed that the PD-MCI group had more cases displaying hyperinnervation compared to PD and PDD groups (Fig. 6b).

### Secondary analysis: galaninergic innervation, cholinergic cell density, Braak staging and demographics

We have also explored further associations regarding galaninergic innervation within our cohort (non-adjusted, two-tailed *p*-values are presented) that may give hints into the role and mechanisms of galaninergic innervation.

**Fig. 7:**
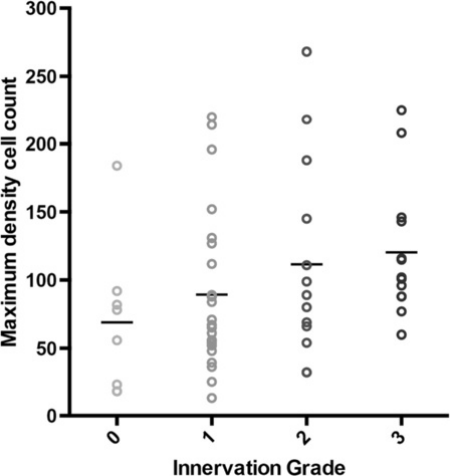
Correlation between innervation scores and maximum density counts of ChAT +ve neurons from adjacent sections (*n* = 60) in Lewy body disease cases. Mean value for every grade is shown with a *horizontal bar*. Spearman’s *rho* = 0.361, *p* = 0.005

Analysis of the LBD cohort revealed a small but significant correlation between innervation grade and maximum density cell count; Spearman’s *rho* = 0.361, *p* = 0.005. See Fig. 7. Maximum cell density count of ChAT +ve neurons from adjacent sections was obtained from on-going projects [34] in our lab (*n* = 60). This trend was preserved after subgroup analysis but did not reach statistical significance probably due to low power (PD: *rho* = 0.398, *p* = 0.066; PD-MCI: *rho* = 0.674, *p* = 0.067; PDD: *rho* = 0.127, *p* = 0.520). Direct comparison of innervation grade 0 (*n* = 8) and grade 3 (*n* = 16) cases showed that in the hyper-innervated cases the mean maximum cell density was nearly twice that of the non-hyper-innervated ones. See Table 2. We also found a negative correlation between galaninergic innervation and Braak tau staging, Spearmans’ *rho* = −0.245, *p* = 0.035; but not with Braak α-synuclein.

**Table 2:** Characteristics of Lewy body disease cases grouped by innervation grade

Innervation Score	Mean age at onset (SE)	Mean age at death (SE)	Mean duration of disease (SE)	Mean ChAT +ve neuron count (SE)*	Median Braak α-synuclein stage	Median Braak tau stage **
0 (*n* = 8)	70.12 (2.546)	78.75 (2.987)	8.63 (1.451)	68.88 (19.59)	6	2
1 (*n* = 33)	63.55 (1.757)	76.61 (1.302)	13.15 (1.085)	89.33 (11.716)	6	2
2 (*n* = 19)	63.42 (1.955)	77.47 (1.885)	14.11 (1.395)	111.62 (20.3)	6	2
3 (*n* = 16)	63.81 (2.802)	75.75 (2.044)	12.13 (1.378)	120.38 (13.581)	6	2

*Spearmans’ rho = *rho* = 0.361, *p* = 0.005. ** *rho* = −0.245, *p* = 0.035

Finally, although the control group was predominantly female, group and subgroup analysis revealed no significant differences between males and females regarding galaninergic innervation. We also found no significant correlations between galaninergic innervation, age at onset, age at death or duration of disease.

## Discussion

### Galaninergic innervation and hyper-innervation

This is the first study to our knowledge that has characterised and semi-quantitatively analysed the immunoreactivity of galanin in the nbM of brains of patients with LBD. The finding of significantly increased innervation density in LBD compared to age-matched controls is in line with previous literature on AD [7, 8, 29]. Yet, here we report that increased innervation or hyper-innervation (grades 2 and 3 innervation) was observed in only half of all LBD cases. Interestingly, the PD-MCI group had the highest proportion of cases displaying grade 3 hyperinnervation although we found no statistically significant differences among the different LBD groups, probably because of the relatively low number of cases. Whether this trend indicates a real activation of the galanin system during the transition from PD to PDD is difficult to know but would be of interest, as Lewy body pathology in the nbM and loss of cholinergic neurons occurs early in PD. In that sense, an inverted U-shape curve would be consistent with an early compensatory reactivity of the galanin system against cognitive dysfunction that may fail in later stages of the disease. In contrast, one similar study [30] assessed galaninergic innervation of anterior nbM neurons in samples of patients with *early* AD, mild cognitive impairment, and no cognitive impairment, but revealed no differences among groups with regards to innervation scores or correlation with cell counts. The authors suggested that hyper-innervation may occur in late rather than early stage AD but a direct comparison between AD and LBD has not been undertaken to our knowledge. Interestingly, in secondary analysis we observed a negative but weak correlation between innervation grade and Braak tau staging. Although this could be a type 1 error (false positive), further examination and quantification of tau, β-amyloid and α-synuclein pathology may elucidate further any potential associations with galanin.

The exact aetiology of galanin fibre plasticity is still largely unresolved but it is thought to relate to local or distant injury of the cholinergic basocortical pathways. Yet, although hyper-innervation may indeed be more common in AD or LBD, the fact that increased innervation occurs only in a subgroup of disease cases implies that it is a secondary reactive phenomenon and not integral to the underlying degenerative processes. Increased GAL-ir fibre density has been observed in the rat basal forebrain after direct excitotoxic lesions of basal cholinergic groups [35, 36] and even ischaemic lesions of cortical target sites [37]. Immunotoxic lesioning of the cholinergic neurons of the horizontal limb of the diagonal band of Broca (Ch3) in the rat with the cholinergic specific 192 IgG-saporin also produces increases in GAL-ir fibre density and thickness that occur as early as one hour and persist for up to 6 months [38]. In these animal studies an increase in fibre density is observed after a single insult, irrespective of the resultant cholinergic cell loss [38]. However, in AD [30] or LBDs, which are progressive, hyper-innervation is not observed in the prodromal or early stages of the disease. Hence, it is still not clear whether hyper-innervation occurs as a direct response to neuronal injury or as part of a feedback mechanism related to the functional status of the cholinergic neurons. Imbalances in excitatory/inhibitory input or output are already known to upregulate galanin in a different paradigm [39].

Furthermore, old rats (20 months old) not only fail to elicit a galanin response to an excitotoxic insult in the nbM compared to young rats, but also show a lower baseline GAL-ir fibre density [35]. Similarly, partial failure of somal galanin upregulation has been observed in the Ch1-Ch2 neurons of old rats after colchicine treatment, which is known to impair fast axonal transport [40]. The underlying reasons for the decreased galanin plasticity in the old rats are not known but would be of great relevance to neurodegenerative conditions and might explain why hyper-innervation is observed only in a subgroup of cases. In our study, however, we found no significant correlation between demographics and extent of innervation.

After secondary analysis within the LBD cohort, we also found a significant correlation between innervation score and ChAT +ve neuronal count and interestingly the 8 cases with very scant galanin fibres (grade 0) not only had a very low cell count, but also a faster disease course and were older age at death compared to the hyper-innervated cases (Table 2). There is already some evidence that galaninergic hyper-innervation in AD is associated with favourable expression of pro-survival mRNAs, as determined by single cell gene expression profiling, and it has also been argued that the caudo-rostral pattern of degeneration of the nbM neurons in AD is related to the reduced galaninergic innervation of the more posterior aspects of the nbM [19, 41]. It would be tempting to consider then that the observed positive correlation is supportive of the neuroprotective role of galanin as suggested previously [19]. However, the design of this study cannot reveal whether such a correlation is causal or whether this just indicates that lower cell density means less available neurons for innervation (*n.b*. this would not hold true for the healthy controls).

### nbM Somal GAL-ir and neuronal injury

Previous literature has been contradictory with regards the presence or not of galanin within the somata of the cholinergic neurons of the nbM. Although there have been previous immunohistochemical observations of *moderate* GAL-ir within the nbM cholinergic neurons of elderly control and AD brains [7, 10, 42, 43] it has also been supported that galanin is expressed by basal cholinergic neurons only in non-human primates and not in the normal or diseased human brain [8, 9, 26, 30, 41, 44]. Similarly, in an RNA hybridization study by Walker et al. [44] there was no co-localization of the galanin RNA probe (directed against bases 228–271 of the rat galanin sequence) and cholinergic neurons in the human nbM, which could be because of the use of a reportedly high threshold. In contrast, in another hybridization study by Chan-Palay et al. [59], using a different probe (directed against bases 324–414 of porcine galanin), mRNA labeling did co-localize with medium-sized nbM neurons and was also slightly increased in AD [45].

In the present study variable perikaryal galanin-like immunoreactivity within magnocellular neurons in the nbM was observed in a number of disease cases. However, although the antibody used is monoclonal and by definition selected and purified for its affinity towards a recombinant human galanin peptide, it is not possible to exclude cross-reactivity with other epitopes and regarding this as intrinsic upregulation of galanin would be still speculative at this point.

**Fig. 8:**
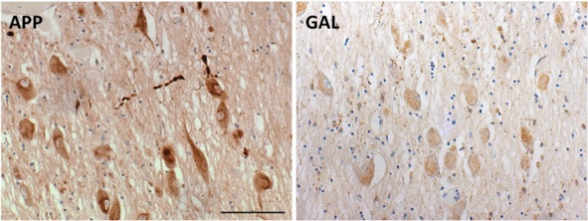
APP immunostaining of nucleus basalis neurons indicating axonal dysfunction in a case associated with perikaryal galanin-like immunoreactivity (GAL; scale bar represents 100 μm)

Nevertheless, a potential upregulation of galanin within the human cholinergic neurons would be consistent with several animal models of neuronal injury: Significant increases in the number of GAL-ir neurons, galanin peptide levels (up to 120-fold) and galanin-mRNA levels have been observed after transection of the rat sciatic nerve [46, 47] or lesions of rat basal cholinergic neurons, their projections and their targets [38, 48, 49]. Upregulation of galanin peptide and mRNA can also be induced by colchicine [43] or tetrodotoxine [49]. All these indicate that physical or functional disruption of axonal homeostasis is sufficient for the reactive upregulation of galanin within the affected cholinergic neurons. Therefore, the observed increases in somal GAL-ir in a subgroup of disease cases might relate to the underlying neuronal injury and may represent endogenous synthesis due to auto-regulation [50]. Following this up, we have preliminary observations that some cases with moderate somal GAL-ir, display somal APP immunoreactivity as well, which is a marker of axonal dysfunction (Fig. 8). The presence or not of galanin within the human cholinergic neurons and its potential relationship with axonal dysfunction will be addressed in future studies.

### Other types of GAL-ir

Finally, the very isolated observation of the putative glia associated with radial GAL-ir (§3.3) in the ventral pallidum is a finding of yet unknown significance and the identity of the associated cells remains unknown. This potential and site-specific association and the observed predominance in PDD cannot be explained and necessitates further validation and investigation.

## Conclusion

This is the first study to provide evidence of increased galanin innervation and possibly somal expression within nbM neurons, in Lewy body disorders without concurrent significant AD pathology. The reason that this response is observed only in a subgroup of disease cases remains rather elusive and this heterogeneity emphasises that galanin upergulation is not an integral part of neurodegeneration but probably a secondary reactive phenomenon. Future research would benefit from inclusion of corroborating techniques that can confidently assess the presence and quantify the levels of galanin mRNA and peptide. Clinicopathological correlations in well-characterised cohorts would then be of importance as well as the direct comparison between LBD and AD. Finally, the development of quantitative approaches is necessary for giving a confident answer to the question of whether basal forebrain galanin upregulation occurs in different neurodegenerative conditions including AD.

### Ethical considerations

Wales Research Ethics Committee approved protocol (Ref. No. 08/MRE09/31+5).

## Additional file

Additional file 1:
**Supplementary information about antibodies and immunostaining comparison.** (DOCX 2924 kb)

## Abbreviations

AD: Alzheimer’s disease
ChAT: Choline-acetyltransferase
DAB: 3′3-diaminobenzidine
DLB: Dementia with Lewy bodies
FFPE: Formalin-fixed paraffin-embedded
GAL-ir: Galanin immunoreactivity
LBD: Lewy body diseases
MCI: Mild cognitive impairment
nbM: Nucleus basalis of Meynert
PBS: Phosphate-buffered saline
PD: Parkinson’s disease
PD-MCI: Parkinson’s disease with mild cognitive impairment
PDD: Parkinson’s disease with dementia
SON: Supraoptic nucleus
