# Supplementary material for: Differential expression of galanin in the cholinergic basal forebrain of patients with Lewy body disorders
Source: Acta Neuropathol Commun. 2015 Dec 1;3:77. doi: 10.1186/s40478-015-0249-4 (PMC4666186; doi:10.1186/s40478-015-0249-4)
Supplement: Additional file 1: — Supplementary information about antibodies and immunostaining comparison. (DOCX 2924 kb) [file 40478_2015_249_MOESM1_ESM.docx]

**SUPPLEMENTARY INFORMATION ABOUT ANTIBODIES AND IMMUNOSTAINING COMPARISON**

RnD antibody (monoclonal mouse anti-human; against E.coli-derived recombinant human Galanin. Ala20-Ser123)

MARGSALLLASLLLAAALSASAGLWSPAKEKRGWTLNSAGYLLGPHAVGNHRSFSDKNGLTSKRELRPEDDMKPGSFDRSIPENNIMRTIIEFLSFLHLKEAGALDRLLDLPAAASSEDIERS

Everest Biotech (Goat anti-human against peptide sequence C-HRSFSDKNGLTSK)

MARGSALLLASLLLAAALSASAGLWSPAKEKRGWTLNSAGYLLGPHAVGNHRSFSDKNGLTSKRELRPEDDMKPGSFDRSIPENNIMRTIIEFLSFLHLKEAGALDRLLDLPAAASSEDIERS

Key:

Black: Signal peptide (1-19)

Blue: Pro-peptide (20-30)

Red: Galanin (33-62)

Green: Galanin message-associated peptide (65-123)

Immunogen for both antibodies denoted by underline

UniProtKB - P22466 (GALA_HUMAN)

**figure S1.** Comparison of galanin-like immunoreactivity of R&D monoclonal galanin antibody(**A)** and Everest polyclonal antibody (**B)** on sections through the anterior nbM from the same case. **C.** Double immunofluorescence of the R&D monoclonal (red) and Everest polyclonal (green) galanin antibodies showing near-complete co-localization (yellow) in galanin fibres within the basal forebrain; wide field fluorescence microscopy, X20.
